# Infliximab biosimilar CT-P13 therapy in patients with Takayasu arteritis with low dose of glucocorticoids: a prospective single-arm study

**DOI:** 10.1007/s00296-018-4159-1

**Published:** 2018-09-18

**Authors:** Eun Hye Park, Eun Young Lee, Yun Jong Lee, You Jung Ha, Wan-Hee Yoo, Byoong Yong Choi, Jin Chul Paeng, Hoon Young Suh, Yeong Wook Song

**Affiliations:** 1 Division of Rheumatology, Department of Internal Medicine, Seoul National University Hospital, 101, Daehak-ro, Jongno-gu, Seoul, South Korea; 2 Division of Rheumatology, Department of Internal Medicine, Seoul National University Bundang Hospital, Seongnam, South Korea; 3 Division of Rheumatology, Department of Internal Medicine, Chonbuk National University, Jeonju, South Korea; 4 Department of Internal Medicine, Seoul Medical Center, Seoul, South Korea; 5 Department of Nuclear Medicine, Seoul National University Hospital, Seoul, South Korea; 6 Department of Molecular Medicine and Biopharmaceutical Sciences, Graduate School of Convergence Science and Technology and College of Medicine, Medical Research Center, Seoul National University, Seoul, South Korea

**Keywords:** Takayasu arteritis, Infliximab, Tumor necrosis factor-alpha, Treatment, Positron emission tomography computed tomography

## Abstract

To evaluate the efficacy and safety of infliximab biosimilar CT-P13 in patients with active Takayasu arteritis (TAK). In this single-center open-label trial, patients with active TAK received CT-P13 at a starting dose of 5 mg/kg at weeks 0, 2, 6, and then every 8 weeks up to week 46. They were followed up until week 54. From week 14 to week 46, patients with inadequate response received increased dose of CT-P13 by 1.5 mg/kg. Concomitant prednisolone was allowed ≤ 10 mg/day. The primary efficacy end point was the achievement of partial or complete remission at week 30. All patients underwent positron emission tomography–computed tomography (PET–CT) at baseline and week 30. Twelve patients with TAK received CT-P13; one patient with protocol violation was excluded from analysis. Nine (81.8%) patients had taken concomitant prednisolone with median dose of 5.0 mg/day. At week 30, three (27.3%) patients achieved complete remission and six (54.5%) patients achieved partial remission. Statistically significant improvements in modified Indian Takayasu Clinical Activity Score (ITAS2010), ITAS-A, and serum levels of erythrocyte sedimentation rate and C-reactive protein were seen at week 30 from baseline. PET parameters were significantly reduced from baseline to week 30, including maximum standardized uptake value, target-to-vein ratio, target-to-liver ratio, and PET Vascular Activity Score. There were no serious adverse events. Treatment with CT-P13 may lead to improvement in clinical, radiographic, and serological activities with lower glucocorticoid requirement in TAK.

**Trial registration number** NCT02457585.

**Electronic supplementary material:**

The online version of this article (10.1007/s00296-018-4159-1) contains supplementary material, which is available to authorized users.

## Introduction

Takayasu arteritis (TAK) is a granulomatous inflammatory vasculitis affecting the aorta and its main branches [1]. Vascular inflammation may cause arterial stenosis, occlusion, dilatation, and aneurysm formation. Corticosteroids (CS) are cornerstones of the initial treatment of TAK [2]. A previous study has estimated that between 46 and 84% of patients require second-line immunosuppressive agents, such as methotrexate (MTX), azathioprine, mycophenolate mofetil, and cyclophosphamide, to achieve and sustain remission with CS dose reduction [3]. Despite that these agents may result in initial remission, relapses remain common when CS is tapered [4]. Therefore, there has been a constant demand for an effective therapeutic option beyond glucocorticoid for TAK.

Tumor necrosis factor α (TNFα) is an attractive therapeutic target in TAK. As a macrophage and T cell product, TNFα is a critical cytokine in granuloma formation [5] as well as in the activation of endothelial cells [6]. TNFα can stimulate macrophages to secrete interleukin (IL)-12 and IL-18 that direct T cells to develop a Th1 phenotype and produce INFγ which induces recruitment of macrophages into tissues [7]. In addition, it has been demonstrated that increased numbers of TNFα-producing T cells are correlated with disease activity [8]. Therefore, TNF inhibition might be an effective strategy for patients with TAK. Several retrospective and observational studies have suggested that TNF inhibitors are effective for treating TAK that is refractory to conventional immunosuppressive therapies [9–13]. However, randomized controlled study on this has not been reported yet.

The lack of accepted and reliable criteria for disease activity is another limitation in clinical research on TAK. Changes in acute phase inflammatory markers do not always reflect disease activity [1]. Erythrocyte sedimentation rate (ESR) and C-reactive protein (CRP) have been shown to be unreliable markers of disease activity, vascular inflammation, and progression [1, 14]. Recently, pentraxin-3 (PTX3) produced by innate immune cells and vascular cells in response to proinflammatory signals [15] has been suggested as a potent biomarker for disease activity in patients with TAK [16, 17]. More recently, human leukocyte antigen-E (HLA-E) as a soluble molecule (sHLA-E) has been proposed as a biomarker for disease activity and course in TAK [18]. ^18^F-fluorodeoxyglucose-positron emission tomography (^18^F-FDG-PET), a functional imaging technique capable of quantifying the burden of inflammation, has been suggested to reflect disease activity in TAK [19, 20].

The objective of this single-center, prospective open-label trial was to evaluate the efficacy and safety of infliximab (IFX) biosimilar CT-P13 in patients with active TAK. We assessed clinical and serological activity along with radiological activity using PET–CT.

## Methods

### Study patients

Patients older than 18 years with newly diagnosed or relapsing active TAK were enrolled. All patients met American College of Rheumatology classification criteria for TAK [21], and had active disease according to the National Institutes of Health criteria [1]. Patients were excluded from study if they had active systemic infections, acute or chronic liver dysfunction, HIV, hepatitis B or C infection, or malignancy. Those who were pregnant or breastfeeding were excluded. Purified protein derivative skin test or QuantiFERON screening for latent TB was required; patients with latent TB could be enrolled if appropriate prophylactic TB treatment was commenced at least 3 weeks before initiation of study.

### Study design

**Fig. 1:**
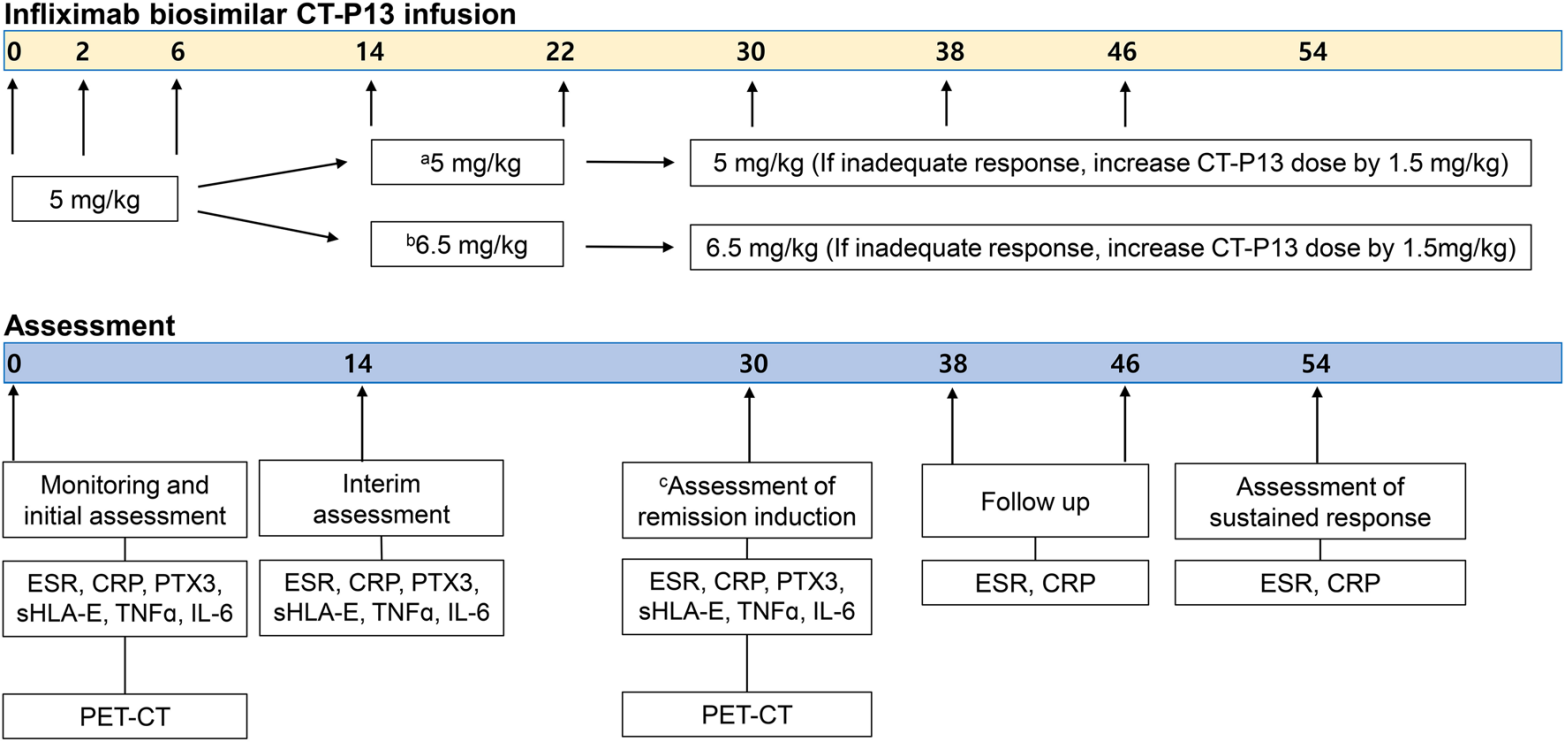
Summary of the study design. **a** Patients whose ESR and CRP improved by ≥ 50% at week 14. **b** Patients whose ESR and CRP did not improve by ≥ 50% or increased. **c** Primary end point. *ESR* erythrocyte sedimentation rate, *CRP* C-reactive protein, *PTX3* pentraxin-3, *sHLA-E* soluble human leukocyte antigen-E, *TNFα* tumor necrosis factor-α, *IL-6* interleukin-6, *ITAS-2010* Indian Takayasu Clinical Activity Score, *PET–CT* positron emission tomography–computed tomography

This is a single-center prospective open-label trial (NCT02457585). The study medication, CT-P13 (Remsima), a biosimilar of IFX was approved by the European Medicines Agency in September 2013 and by the U.S. Food and Drug Administration in April 2016 for all indications of the originator product [22, 23]. Patients who had taken a daily dose of 10 mg of prednisolone (or its equivalent) or lower were allowed to enter the study. The dose was maintained for at least 2 weeks prior to the initiation of CT-P13 and throughout the study. The use of concomitant immunosuppressive agents was permitted with stable doses from 6 weeks prior to the initiation of CT-P13 through the end of the study. Patients received intravenous infusions of CT-P13 at a starting dose of 5 mg/kg at weeks 0, 2, and 6, and then every 8 weeks up to week 46 (Fig. 1). Patients whose levels of ESR and CRP had not improved by ≥ 50% at week 14 were required to increase the CT-P13 dose by 1.5 mg/kg to 6.5 mg/kg. At week 30, patients with complete remission maintained their current CT-P13 dose while patients with partial remission received increased CT-P13 dose by 1.5 mg/kg. Patients who failed with CT-P13 at week 30 terminated the study. At weeks 38 and 46, patients whose symptoms of active disease did not resolve or whose levels of ESR and CRP did not decline to normal values were instructed to increase the dose of IFX by 1.5 mg/kg up to 9.5 mg/kg at each point. Patients were followed up to week 54.

This study was conducted in accordance with ethical principles of the Declaration of Helsinki and Good Clinical Practice guidelines. The study protocol was approved by the Institutional Review Board of Seoul National University Hospital (IRB No: 1312-079-541). All patients provided written informed consent before the first study procedure. Patients were enrolled from March 2013 to September 2017.

### Study assessments

The primary efficacy end point was the achievement of partial or complete remission at week 30. Secondary end points included changes in modified Indian Takayasu Clinical Activity Score (ITAS2010), ITAS-A (ITAS2010 plus scores for ESR/CRP), and serum levels of acute phase reactants at week 30 from baseline. For the original ITAS2010, only new items (occurring or worsening during the previous 3 months) are supposed to be scored at follow-up [24]. Therefore, it may lead to high score only at the initial visit with extremely low scores during follow-up visits even when patients had persistent symptoms without any improvements throughout follow-up. Hence, in order to accurately assess the effect of CT-P13, we modified it to score all positive items present at follow-up visits. Additional efficacy measures included improvements of vascular inflammation by PET–CT and reductions in levels of serologic parameters including PTX3, sHLA-E, IL-6, and TNFα at week 30.

Complete remission for the purpose of this study had to fulfill all of the following: (1) resolution of symptoms of active disease, (2) decline of levels of ESR and CRP to normal values, and (3) decrease of maximum standardized uptake values (SUVmax) of ^18^F-FDG in arterial walls at week 30. Patients were considered as having partial remission if they satisfied two of the above three criteria. Otherwise they were classified as study failure. Clinical and serological responses at week 54 were defined as resolution of symptoms of active disease, and decline of ESR and CRP levels to normal values, respectively.

Clinical laboratory tests, modified ITAS2010, vital signs, and other safety assessments were performed at scheduled visits. Occurrence and severity of all adverse events (AEs) were recorded.

### FDG-PET acquisition and analysis

After fasting for more than 6 h, 5.18 MBq/kg of FDG was administered intravenously and images were acquired 60 min later using PET/CT scanners (Biograph40 or Biograph64, Siemens Healthcare). After CT scan, PET image was acquired for torso area (from skull to the proximal thigh) or whole body. FDG-PET scan images were reviewed by two independent nuclear physicians, who were blinded to the clinical disease activity corresponding to each scan. In cases of disagreement, the final interpretation was determined by consensus between the two nuclear physicians after an additional review. For visual analysis, arterial FDG uptake was evaluated in 11 arterial regions: ascending aorta, aortic arch, right innominate artery, bilateral subclavian arteries, bilateral common carotid arteries, descending thoracic aorta, abdominal aorta, and bilateral common iliac arteries. In each region of interest (ROI), uptake was scored using a 4-point scale (0 = no abnormal uptake, 1 = less than liver uptake, 2 = similar to liver uptake, and 3 = greater than liver uptake). PET Vascular Activity Score (PETVAS), a qualitative summary score based on global arterial FDG uptake visually assessed in specified nine arterial territories, excluding bilateral common iliac arteries from the above mentioned 11 regions, was also evaluated using a 4-point scale.

For quantitative analysis, ROI was drawn for each of 11 arterial regions. In each ROI, SUVmax was measured as the index for the highest inflammatory activity after removing physiologic or specific organ uptake. Target-to-liver ratio (TLR) was acquired by dividing SUVmax of arteries with mean SUV of the liver. Target-to-vein ratio (TVR) was obtained by dividing SUVmax of arteries with mean SUV of veins.

### Serologic parameters

Serum levels of PTX3, sHLA-E, interleukin (IL)-6, and TNFα were measured at baseline visit, week 14, and week 30 in all subjects. Serum concentrations of PTX3 were evaluated with a sandwich enzyme-linked immunosorbent assay (ELISA) (Enzo Life Sciences International, Plymouth Meeting, PA, USA) [25]. Serum sHLA-E concentrations were quantified using an ELISA kit (LifeSpan BioSciences, Seattle, WA, USA) according to the manufacturer’s protocol. IL-6 and TNFα concentrations in serum samples were analyzed by Luminex assay (Millipore, Billerica, MA, USA). Samples were run in duplicates.

### Statistical analysis and sample size

Remission induction rate in TAK patients treated with CT-P13 was estimated to be 90% based on prior published literature [26]. Sample size estimation determined that seven patients would provide 80% power for comparison between anti-TNF therapy and CS therapy in remission induction rate (assumed 90% vs. 50%, respectively) at week 30. Considering 50% of potential dropout rate conservatively, adjusted sample size obtained was 11. Data are presented as mean ± standard deviations (SD) or medians with interquartile ranges (IQRs) for continuous variables and frequencies with percentages for categorical variables. Chi-square test or Fisher’s exact test was used to compare categorical variables while non-parametric Mann–Whitney *U* test or Wilcoxon signed-rank test was used to compare continuous variables as appropriate. Statistical significance was considered at *p* < 0.05. All statistical analyses were performed using SPSS version 23 (SPSS Inc., Chicago, IL, USA) and GraphPad Prism version 5.1 (GraphPad Software, San Diego, CA, USA, 2007).

## Results

### Patient characteristics

**Fig. 2:**
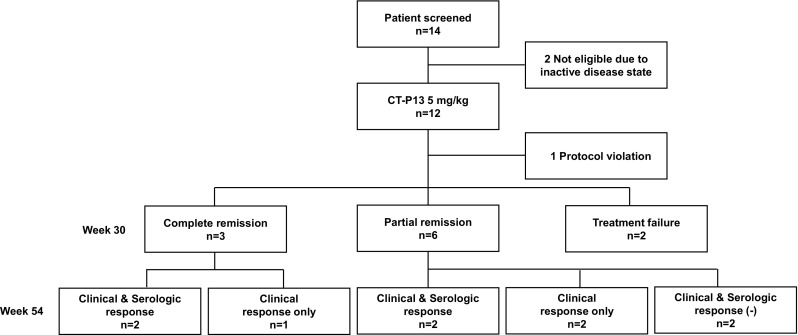
Patient disposition through week 54 and outcome of treatment of patients with Takayasu arteritis

A total of 14 patients with TAK were screened and 12 patients received IFX biosimilar CT-P13 (Fig. 2). Two patients were excluded due to inactive disease state, and one patient with protocol violation was excluded from analysis. All study subjects (*n* = 11) were females with a mean age of 46.8 years (± SD 13.5 years) (Table 1). Mean age at onset of TAK was 42.5 years (± SD 14.8 years). Mean disease duration prior to CT-P13 therapy was 4.4 years (± SD 5.2 years). The majority of patients (90.9%) had experienced multiple relapses while only one patient was newly diagnosed. Patients with active disease were included, with median ITAS2010 and ITAS.A (ESR) at baseline of 11.0 (IQR 10.0–11.8) and 13.5 (IQR 12.0–14.0), respectively. Nine (81.8%) patients received concomitant CS (median 5.0 mg/day of prednisolone) and eight (72.7%) patients received concomitant immunosuppressive agents (Supplementary Table 1**)**. Supplementary Table 2 demonstrates each patient’s demographic and clinical characteristics.

**Table 1:** Baseline clinical and demographic features of 11 patients with Takayasu arteritis

	Infliximab (*n* = 11)
Patient demographics	
Age at enrollment, mean ± SD, years	46.8 ± 13.5
Age at diagnosis, mean ± SD, years	42.5 ± 14.8
Female, *n* (%)	11 (100.0)
Diagnosis category at enrollment, *n* (%)	
Newly diagnosed	1 (9.1)
Relapsing	10 (90.9)
Disease duration, mean ± SD, years	4.4 ± 5.2
BMI, mean ± SD, kg/m^2^	24.9 ± 2.9
Smoking, *n* (%)	0 (0.0)
Comorbidities, *n* (%)	
Hypertension	9 (81.8)
Diabetes mellitus	1 (9.1)
Stroke	2 (18.2)
Coronary artery disease	3 (27.3)
Valvular heart disease	1 (9.1)
Peripheral artery disease	3 (27.3)
Dyslipidemia	3 (27.3)
Heart failure	2 (18.2)
Clinical manifestation at enrollment, *n* (%)	
Headache	3 (27.3)
Extremity claudication	6 (54.5)
New vascular stenosis or aneurysm	6 (54.5)
Vascular pain	3 (27.3)
Musculoskeletal symptoms	5 (45.5)
Ischemic retinopathy	1 (9.1)
Laboratory tests, median (IQR)	
White blood cell, /mm^3^	9610.0 (8705.0–11450.0)
Hemoglobin, g/dL	12.6 (11.3–13.1)
Platelet, /mm^3^	367.5 (297.3–459.5)
ESR, mm/hr	68.5 (38.5–107.3)
CRP, mg/L	1.4 (1.0–4.7)
Serum albumin, g/dL	4.0 (3.9–4.3)
BUN, mg/dL	13.0 (10.8–15.5)
Creatinine, mg/dL	0.7 (0.6–0.8)
Serum pentraxin-3, pg/ml	3782.6 (3230.1–5218.5)
Serum sHLA-E, pg/ml	262.0 (122.7–323.9)
Serum IL-6, pg/ml	5.6 (4.7–10.2)
Serum TNFα, pg/ml	2.9 (2.7–3.8)
Baseline disease activity, median (IQR)	
ITAS2010	11.0 (10.0–11.8)
ITAS-A (ESR)	13.5 (12.0–14.0)

*SD* standard deviation, *IQR* interquartile range, *BMI* body mass index, *ESR* erythrocyte sedimentation rate, *CRP* C-reactive protein, *BUN* blood urea nitrogen, *sHLA-E* soluble human leukocyte antigen-E, *IL-6* interleukin-6, *TNFα* tumor necrosis factor-α, *ITAS2010* Indian Takayasu Clinical Activity Score, *ITAS-A* ITAS2010 plus scores for ESR/CRP

### Clinical responses and efficacy assessments

At week 30, three (27.3%) of the 11 patients treated with CT-P13 achieved complete remission and six (54.5%) patients achieved partial remission (Fig. 2). In two patients, CT-P13 therapy failed. Sustained clinical and serologic response was observed through week 54 in four (44.4%) of the nine patients that initially achieved remission (complete or partial remission) with CT-P13. The other three (33.3%) maintained clinical response but had abnormalities of acute phase reactants. An increase in CT-P13 dosage was required to sustain clinical response, with mean CT-P13 dose of 7.1 mg/kg (median: 8.0 mg/kg, IQR: 5.8–8.0 mg/kg) every 8 weeks. In two patients who had neither clinical nor serological response at week 54, the dose of CT-P13 was mistakenly not increased at week 38 or 46. Therefore, different responses might have been obtained if they were given an increased dose of CT-P13.

**Fig. 3:**
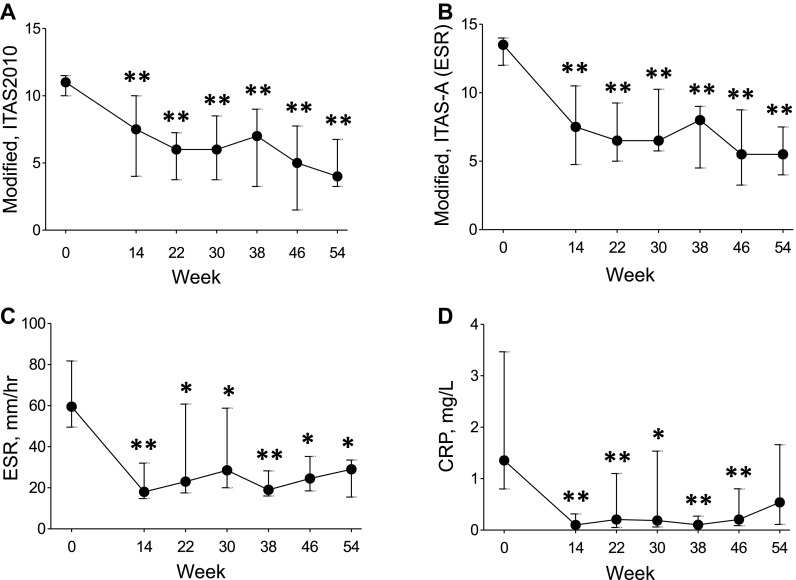
Modified ITAS2010 (**a**), ITAS-A (ESR) (**b**), ESR (**c**), and CRP (**d**) in Takayasu arteritis (*n* = 11). Values are medians and IQRs. *ITAS2010* Indian Takayasu Clinical Activity Score, *ESR* erythrocyte sedimentation rate, *CRP* C-reactive protein. *p ≤ 0.05, **p ≤ 0.01 vs. baseline (Wilcoxon signed-rank test)

Statistically significant improvements were seen at week 30 for all major secondary measures, including change from baseline in modified ITAS2010 [median and IQR at baseline and week 30, 11.0 (10.0–11.8) vs. 6.0 (5.0–9.0), *p* = 0.004], modified ITAS-A (ESR) [14.0 (12.0–14.0) vs. 7.0 ( 6.0–10.5), *p* = 0.003], and serum levels of ESR [56.0 (44.0–82.5) vs. 26.0 (20.0–56.5), *p* = 0.031] and CRP [1.3 (0.7–2.6) vs. 0.2 (0.1–2.1), *p* = 0.019] (Fig. 3). Statistically significant reduction from baseline to weeks 38, 46, and 54 was also seen in modified ITAS2010, ITAS-A (ESR), and serum ESR level. CRP level was significantly decreased at weeks 38 and 46.

**Fig. 4:**
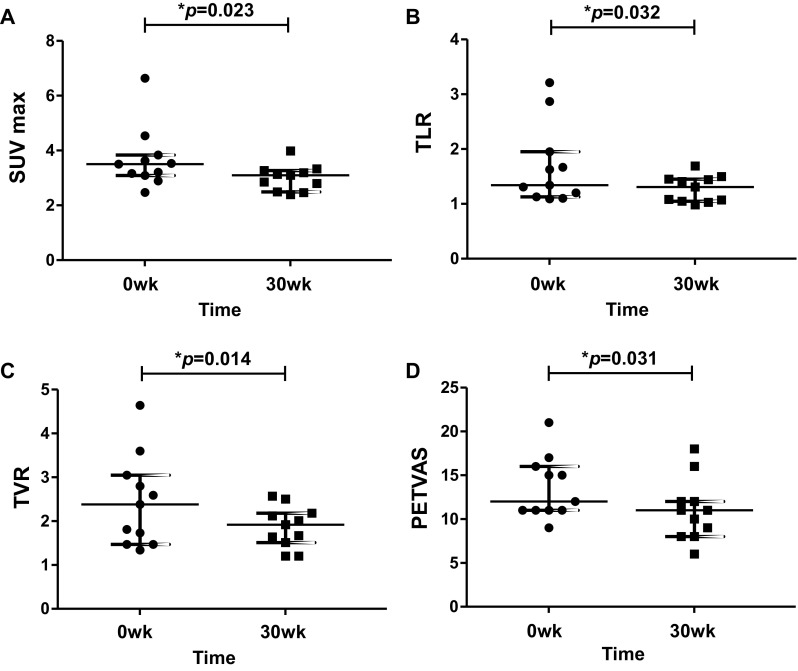
Measurement of ^18^F-FDG uptake on PET–CT before and after anti-TNF therapy in patients with Takayasu arteritis. Horizontal bars represent median and IQRs. **a** Changes from baseline in SUVmax values of ^18^F-FDG in arterial walls. **b** Changes from baseline in values of TVR defined as the ratio of SUVmax of a vascular ROI to the mean SUV of vein. **c** Changes from baseline in values of TLR defined as the ratio of SUVmax of a vascular ROI to the mean SUV of liver. **d** Changes from baseline in PETVAS, a summary score based on global arterial FDG uptake visually assessed in specified nine arterial territories (ascending, arch, descending thoracic and abdominal aorta, innominate, carotid, and subclavian arteries) using a 4-point scale. ^*18*^*F-FDG* ^18^F-fluorodeoxyglucose, *PET–CT* positron emission tomography–computed tomography, *SUV*_*max*_ maximum standardized uptake value, *TVR* target-to-vein ratio, *ROI* region of interest, *TLR* target-to-liver ratio, *PETVAS* PET vascular activity score

All patients showed active vascular ^18^F-FDG uptake (visual grade ≥ 2) in at least 1 vascular region at baseline PET–CTs. The median number of vascular regions with target-to-liver ratio (TLR) greater than 1 was 6 (IQR: 3.0–7.5) and median SUVmax was 3.5 (IQR: 3.1–3.8) at baseline. Quantitative ^18^F-FDG-PET parameters were significantly reduced from baseline to week 30, including SUVmax (3.50, IQR: 3.10–3.84 vs. 3.10, IQR: 2.49–3.27, *p* = 0.023), TVR (1.34, IQR: 1.13–1.95 vs. 1.31, IQR: 1.05–1.45, *p* = 0.032), and TLR (2.38, IQR: 1.47–3.05 vs. 1.92, IQR: 1.51–2.18, *p* = 0.014) (Fig. 4). Statistically significant reduction in PETVAS from baseline to week 30 was also seen (12.0, IQR: 11.0–15.5 vs. 11.0, IQR: 8.0–12.0, *p* = 0.031).

Overall, serum median levels of PTX3, sHLA-E, IL-6 tended to be decreased while TNFα level was significantly increased after CT-P13 therapy (Supplementary Fig. 1). Serological parameters in patients with remission and non-remission were analyzed. At baseline, serum levels of TNFα were significantly lower in patients with remission compared to those in patients without remission (*p *= 0.036). Levels of PTX3, sHLA-E, and IL-6 also tended to be lower in those with remission (Supplementary Fig. 2**)**.

### Adverse events

During the treatment period, there were no serious adverse events (SAEs) or AEs necessitating discontinuation of CT-P13. One patient who had a mild infusion reaction to CT-P13 was premedicated with acetaminophen. Minor infection events were the most frequently reported AEs, including upper respiratory tract infection and viral keratitis (Table 2). At screening, one patient was diagnosed with latent TB infection by a positive QuantiFERON. No event of active TB infection occurred during the follow-up. Other events judged to be possibly related to CT-P13 included dizziness, non-cardiac chest pain, and edema.

**Table 2:** Adverse events of infliximab in patients with Takayasu arteritis (*n* = 12)

Adverse events	*n* (%)
Infection	4 (33.3)
Upper respiratory tract infection	3 (25.0)
Viral keratitis	1 (8.3)
Dizziness	2 (16.7)
Edema	2 (16.7)
Chest pain, non-cardiac	1 (8.3)
Infusion reaction	1 (8.3)

## Discussion

This is the first prospective open-label trial of anti-TNF therapy in patients with TAK using PET–CT to assess vascular inflammation. It was designed to evaluate the efficacy and safety of IFX biosimilar CT-P13 with concomitant prednisolone use of 10 mg/day or less. Results suggested that CT-P13 might lead to remission or improvement with lower CS requirement in TAK. Nine patients (81.8%) had complete or partial remission at week 30 and seven (77.8%) patients had sustained clinical response with or without serological response at week 54. Among these patients, two patients had neither sustained clinical nor serological response at week 54. These patients had high levels of ESR and CRP at weeks 38 and 46, thus requiring increased dose of CT-P13 by 1.5 mg/kg at each point. However, they were not administered with increased dose of CT-P13 mistakenly from week 38. Therefore, sustained response might have been obtained if they had been given an increased dose of CT-P13 as planned. Overall, CT-P13 led to significant improvements in clinical disease activities as well as radiographic and serological activities.

Our results are concordant with those of previous studies. In multiple retrospective observational studies, anti-TNF therapy has been reported to be effective in the majority of refractory TAK patients [9, 10, 26]. A summary of observational studies including 120 patients with refractory TAK receiving anti-TNF agents has shown a response rate of 80% and CS tapering effect in more than 40% of patients [7]. However, relapses occurred in 37% of patients and nearly half of relapsing patients required either an increase in dose or frequency or a switch to different anti-TNF agents (37). In this summary, IFX was used in the majority (80%) of patients while the remaining patients had used adalimumab (ADA) or etanercept (37). In another case series of nine active TAK patients resistant to conventional immunosuppressive agents, proportions of patients reaching complete remission and partial remission with anti-TNF therapy were 56% and 34%, respectively (31). For all patients, CS could be reduced to less than 10 mg/day of prednisone or its equivalent [23]. However, nearly half of patients relapsed following increasing dose intervals of anti-TNF agents [31].

The dose and frequency of IFX infusion varied among studies since there is no current guideline for anti-TNFs in TAK. Several authors have reported that attempts to administer IFX 3 mg/kg every 8 weeks are unsuccessful [27, 28]. In the present study, patients initially received IFX biosimilar CT-P13 at a dose of 5 mg/kg, followed by the same sequential regimen as used in ankylosing spondylitis (AS). However, CT-P13 dose was required to be increased, with mean dose of 7.1 mg/kg every 8 weeks, to obtain clinical response. This is higher than the standard dose of CT-P13 used in treatments of rheumatoid arthritis (RA) and AS. Nevertheless, for patients with RA who have an incomplete response with CT-P13, it is allowed to adjust the dose up to 10 mg/kg, which is higher than the mean dose of CT-P13 used in this study. Taking account of adverse events associated with high dosage of CT-P13, such as increased risk of infections, the dose or frequency of CT-P13 should be adjusted according to each patient’s clinical settings. Further studies are in need to provide precise treatment guidelines for IFX or CT-P13 in patients with TAK.

Anti-TNF agents have demonstrated efficacy in treatment of TAK refractory to other therapies. Nevertheless, the utility of anti-TNF agents in newly diagnosed patients with TAK is yet unknown [29]. With this regard, Serra et al. [30] have reported five newly diagnosed patients treated with anti-TNF therapy in an open-label parallel group: IFX + MTX (*n* = 2) vs. ADA + MTX (*n* = 2) vs. ADA alone (*n* = 1). All five patients responded and achieved clinical and laboratory remission [30], suggesting the efficacy of anti-TNF agents early in the disease course [29]. Our study included only one patient newly diagnosed with TAK. This patient had partial remission at week 30 with sustained clinical response at week 54. However, it is largely limited to discuss about the efficacy of IFX in newly diagnosed patients. Further studies are needed to clarify whether anti-TNFs could be used earlier in the treatment of TAK.

Among biologic treatment options other than anti-TNF agents, tocilizumab (TCZ) is a promising agent for the treatment of TAK as IL-6 is also known to have a critical role in the pathogenesis of TAK [30]. Several studies on TCZ treatment in relapsing/ refractory TAK patients have been reported [31, 32]. Recently, a randomized, double-blind, placebo-controlled, phase 3 trial of TCZ in patients with TAK has suggested that there is no significant difference in time to relapse between tocilizumab and placebo groups [33]. A randomized, double-blind trial of abatacept for treatment of TAK has demonstrated that the addition of abatacept to a treatment regimen with prednisone does not reduce the risk of relapse [34].

Assessment of disease activity in TAK patients remains challenging. ESR is widely used, but the values are reported to be normal in 30% of patients with active inflammation and elevated in nearly half of patients in clinical remission [1, 35]. In a single-center study from Italy, PTX3 levels were found to be higher in active TAK patients than those with inactive disease and healthy controls (23). Plasma PTX3 level greater than 1 ng/mL [area under the curve (AUC) 0.919, 95% CI 0.847–0.991] was shown to be more accurate than normal thresholds of ESR or CRP for distinguishing active from inactive disease [17]. sHLA-E levels were also higher in active TAK than in those in stable disease [24]. At an optimal cut off levels of 21.9 pg/ml, sHLA-E could differentiate active from stable disease, with a sensitivity of 82.6%, specificity of 77.8%, and an AUC of 0.81 (95% CI 0.66–0.95) [18]. ^18^FDG-PET is an imaging tool considered as a reliable method for evaluating vascular inflammation [19, 20]. A meta-analysis of six studies has reported that ^18^F-FDG-PET has moderate diagnosis value in assessing disease activity, with a sensitivity of 70.1% and a specificity of 77.2% [36].

All patients in the current study had high disease activity at baseline, with high ITAS 2010 and ITAS.A (ESR), active vascular ^18^F-FDG uptake, and high ESR and CRP at baseline. After treatment with CT-P13, significant decreases in modified ITAS2010, ITAS-A (ESR), and levels of ESR and CRP, as well as declining tendencies of PTX3 and sHLA-E were found. PET parameters including SUVmax, TLR, TVR, and PETVAS were significantly reduced from baseline to week 30. Consistent with previously reported results [37], IL-6 tended to decrease while TNFα level was increased after CT-P13 therapy. Since IFX as an anti-TNFα antibody only binds to TNFα, not to its receptor [38], the mechanism through which IFX elevated serum TNFα level was difficult to explain, despite improvements of active symptoms and serum ESR and CRP levels. This may be in line with the context of the study of Takeshita et al. [39] which suggests that evaluation of TNFα level after IFX therapy is difficult due to interference from drug–cytokine complexes [37]. However, patients with remission had significantly lower serum levels of TNFα at baseline compared to those with non-remission. Interestingly, patients with remission tended to have lower serum levels of PTX3, sHLA-E, and IL-6 at baseline (Supplementary Fig. 2).

Some of the patients were over 40 years of age at diagnosis of TAK; distinction between TAK and giant cell arteritis (GCA) was made based upon the distribution of vascular lesions and the ethnic origin, given that GCA is a rare disease among Asians. The baseline CT angiography revealed involvements of the aortic arch, thoracic and abdominal aorta, common carotid, subclavian, mesenteric, renal and iliac arteries (Supplementary Table 2). Although imaging modalities such as color duplex ultrasonography and high-resolution contrast-enhanced magnetic resonance imaging of the temporal arteries [40] were not performed in the patients, none of the patients demonstrated scalp tenderness, decreased pulsation of temporal artery, or jaw claudication, which are common in GCA.

This is the first-ever prospective trial of anti-TNF therapy, which includes analysis of ^18^FDG-PET in patients with TAK. IFX biosimilar CT-P13 exhibited improvements in clinical, serological, and radiographic features, even in the setting of low dose of CS. We measured FDG uptake using various quantitative methods, including SUVmax, TLR, TVR and MTV. Vascular inflammation decreased after CT-P13 therapy, suggesting that PET not only reflects active disease but also decrease of inflammation after therapy.

This study has several limitations. As this was an open-label trial with relatively small number of patients, definite conclusions concerning the efficacy of IFX in TAK could not be reached. Due to ethical limitations, the study did not include a control group. A larger randomized controlled trial is warranted to substantiate findings of this study. In addition, the duration of the study period (54 weeks) was not long enough to confirm the long-term duration of remission or safety of CT-P13. Another limitation lies in the definition of remission in this study. We set reduction of ESR and CRP to normal values as one of the criteria for remission. However, an elevation of acute phase reactants does not necessarily reflect disease activity in the absence of clinically compatible disease manifestations. Moreover, the lack of PET–CT at the end of the study at week 54 is another limitation that should be mentioned. We could not evaluate whether improvements of vascular inflammation observed with PET–CT at week 30 sustained until week 54, while clinical and serological responses sustained through week 54.

In summary, this prospective open-label trial of IFX biosimilar CT-P13 suggests that CT-P13 therapy may lead to remission or improvement with lower glucocorticoid requirement in TAK. Randomized controlled studies are warranted to assess the long-term efficacy and safety of IFX and propose precise treatment guidelines for IFX in TAK.

## Electronic supplementary material

Below is the link to the electronic supplementary material.

Supplementary material 1 (DOCX 178 KB)
