# Supplementary material for: Infliximab biosimilar CT-P13 therapy in patients with Takayasu arteritis with low dose of glucocorticoids: a prospective single-arm study
Source: Rheumatol Int. 2018 Sep 18;38(12):2233–42. doi: 10.1007/s00296-018-4159-1 (PMC6223861; doi:10.1007/s00296-018-4159-1)
Supplement: Supplementary file 1 — Supplementary material 1 (DOCX 178 KB) [file 296_2018_4159_MOESM1_ESM.docx]

**Supplementary materials**

**Infliximab therapy in patients with Takayasu arteritis: A prospective single arm study**

Eun Hye Park, Eun Young Lee, Yun Jong Lee, You Jung Ha, Wan-Hee Yoo, Byoong Yong Choi, Jin Chul Paeng, Hoon Young Suh, Yeong Wook Song.

**Supplementary Table I. Concomitant immunosuppresive agents in 8 patients with Takayasu arteritis during study period (54 weeks)**

**Supplementary Table 2. Demographic and clinical characteristics of each patient (n=11) with Takayasu arteritis**

**Supplementary Fig. 1 Changes of serum levels of PTX3 (A), sHLA-E (B), IL-6 (C), and TNFα (D) in Takayasu arteritis (n = 11) from baseline.**

**Supplementary Fig. 2 Serum levels of PTX3, sHLA-E, IL-6, and TNFα in patients with remission (n = 9) and non-remission (n = 2) at baseline and week 14.**

**Supplementary Table 1. Concomitant immunosuppresive agents in 8 patients with Takayasu arteritis during study period (54 weeks)**

| Agent | N (%) | Mean dose | Dose, median (IQR) |
| --- | --- | --- | --- |
| Methotrexate | 8 (72.7) | 16.3 mg/week | 15.0 mg/week (14.4-18.1) |
| Azathioprine | 2 (18.2) | 112.5 mg/day | 112.5 mg/day  (81.3-143.8) |
| Hydroxychloroquine | 1 (9.1) | 400.0 mg/day | 400 mg/day |

**Supplementary Table 2. Demographic and clinical characteristics of each patient (n=11) with Takayasu arteritis**

| **Patient** | **Age at enrollment, years** | **Age at Dx., years** | **Sex** | **Disease duration, years** | **Vascular lesions at enrollment** | **Immunossuppressive therapies prior to CT-P13 treatment** | | **Concomitant immunosuppresive therapies during CT-P13 treatment** | | **Treatment response** | | **Final dose of CT-P13, mg/kg** |
| --- | --- | --- | --- | --- | --- | --- | --- | --- | --- | --- | --- | --- |
|  |  |  |  |  |  | **PD**^a^**, mg/day** | **Others** | **PD, mg/day** | **Others** | **Week 30** | **Week 54** |  |
| 1 | 60 | 60 | F | 0 | Aneurysmal dilatation of descending thoracic and suprarenal abdominal aorta | 0 | None | 0 | None | PR | Clinical response only | 8 |
| 2 | 53 | 52 | F | 1.2 | Wall thickening from aortic root to distsal thoracic aorta; stenosis of left SCA; aneurysmal dilatation of the ascending thoracic aorta; AR | 7.5 | MTX | 2.5 | MTX | CR | Clinical & serologic response (+) | 5 |
| 3 | 56 | 54 | F | 2.1 | Occlusion in Right SCA; left SCA anurysmal dilatation; stenosis of bilateral CCAs and SMAs; occlusion in bilateral IIAs | 10 | MTX, AZA | 7.5 | None | PR | Clinical & serologic response (-) | 6.5 |
| 4 | 51 | 43 | F | 8.5 | Stenosis in bilateral pulmonary arteries; wall thickening of descending thoracic and abdominal aorta | 15 | MTX, AZA | 5 | MTX | CR | Clinical response only | 5 |
| 5 | 21 | 15 | F | 6.5 | Dilated thoracic aorta; stenosis in pulmonary arteries and abdominal aorta | 5 | MTX, AZA,  oral CYC | 2.5 | MTX, AZA | PR | Clinical & serologic response (-) | 6.5 |
| 6 | 53 | 39 | F | 14.6 | Aortic dissection, Stanford type B; occlusion in right SCA; stenosis in left SCA; wall thickening of aortic arch and infrarenal abdominal aorta | 10 | AZA | 5 | None | PR | Clinical & serologic response (+) | 6.5 |
| 7 | 23 | 16 | F | 7.0 | Aortic root aneurysm; dilated ascending aorta; wall thickening of both CCAs, left SCA and thoracoabdominal aorta | 15 | MTX, MMF, CYC pulse | 10 | MTX | Failure |  | 6.5 |
| 8 | 28 | 27 | F | 0.6 | Aortic arch wall thickening; stenosis in left CCA | 7.5 | MTX | 5 | MTX | PR | Clinical & serologic response (+) | 9.5 |
| 9 | 50 | 49 | F | 0.8 | Wall thickening of descending thoracic and abdominal aorta, bilateral CIAs, EIAs and IIAs; occlusion in left SCA and SMA | 7.5 | MTX | 2.5 | MTX | CR | Clinical & serologic response (+) | 8 |
| 10 | 56 | 37 | F | 19.6 | Aorta to left CCA bypass; 70% stenosis of proximal graft; occlusion in both CCAs, right ICA, both SCAs, axillary artery, SMA and IMA; 2 stents inserted in right renal artery with stenosis in proximal os | 20 | MTX, AZA | 7.5 | MTX, AZA | PR | Clinical response only | 8 |
| 11 | 51 | 48 | F | 3.7 | Aortic arch wall thickening; stenosis in descending aorta | 5 | MTX | 0 | MTX | Failure |  | 6.5 |

a The lowest effective dosage without relapse

Dx, diagnosis; SCA, subclavian artery; AR, aortic regurgitation; CCA, common carotid artery; SMA, superior mesenteric artery; IIA, interal iliac artery; CIA, common iliac artery; EIA, external iliac artery; ICA, internal carotid artery; IMA, inferior mesenteric artery; PD, prednisolone; MTX, methotrexate; AZA, azathioprine; CSA, cyclosporine; CYC, cyclophosphamide; MMF, mycophenolate mofetil; PR, partial remission; CR, complete remission.

**Supplementary Figures**

**
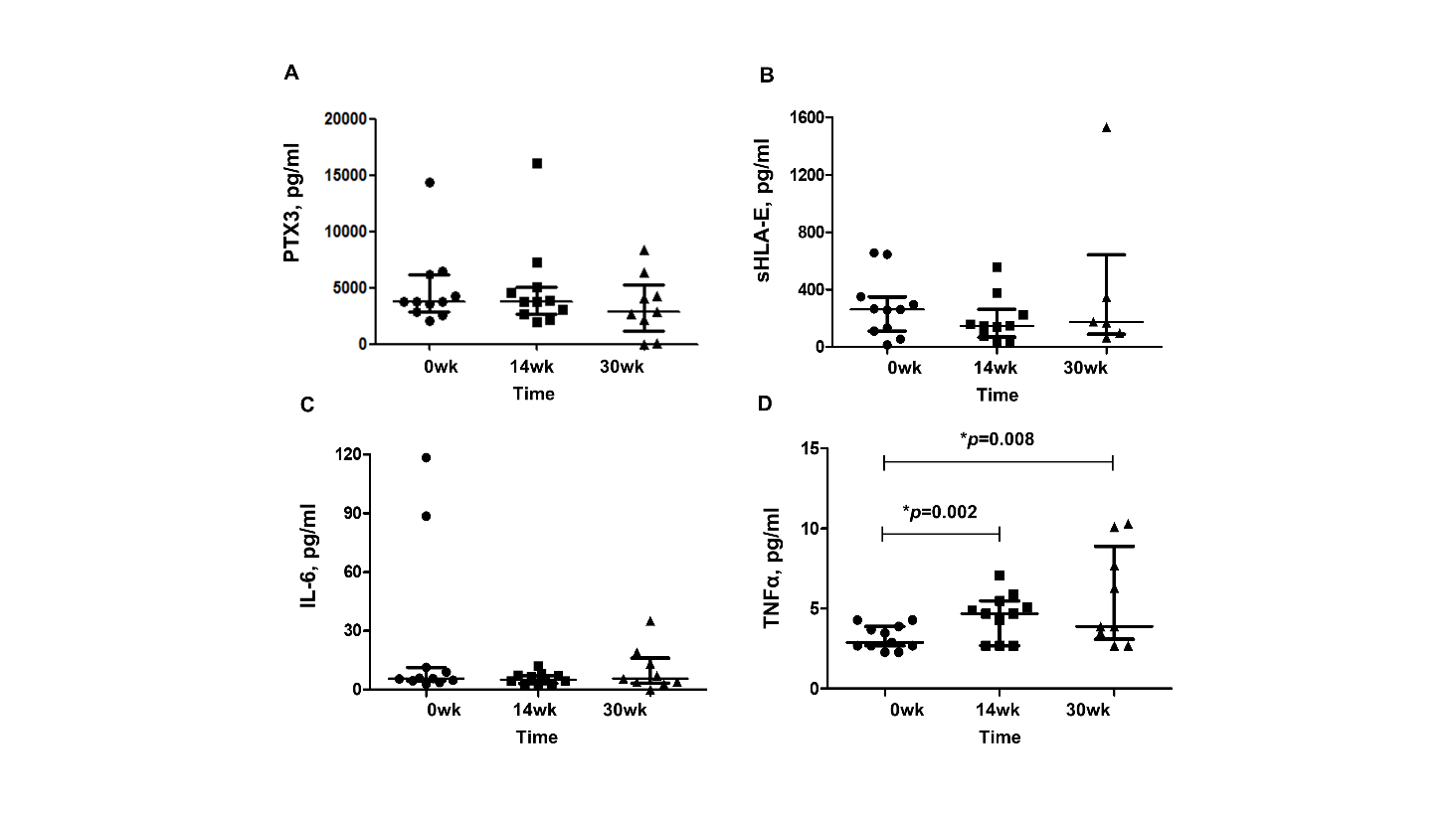
**

**Fig. 1 Changes of serum levels of PTX3 (A), sHLA-E (B), IL-6 (C), and TNFα (D) in Takayasu arteritis (n = 11) from baseline.** Values are median and IQRs. PTX3, pentraxin 3; sHLA-E, soluble human leukocyte antigen-E; IL-6, interleukin-6; TNFα, tumor necrosis factor-α

**
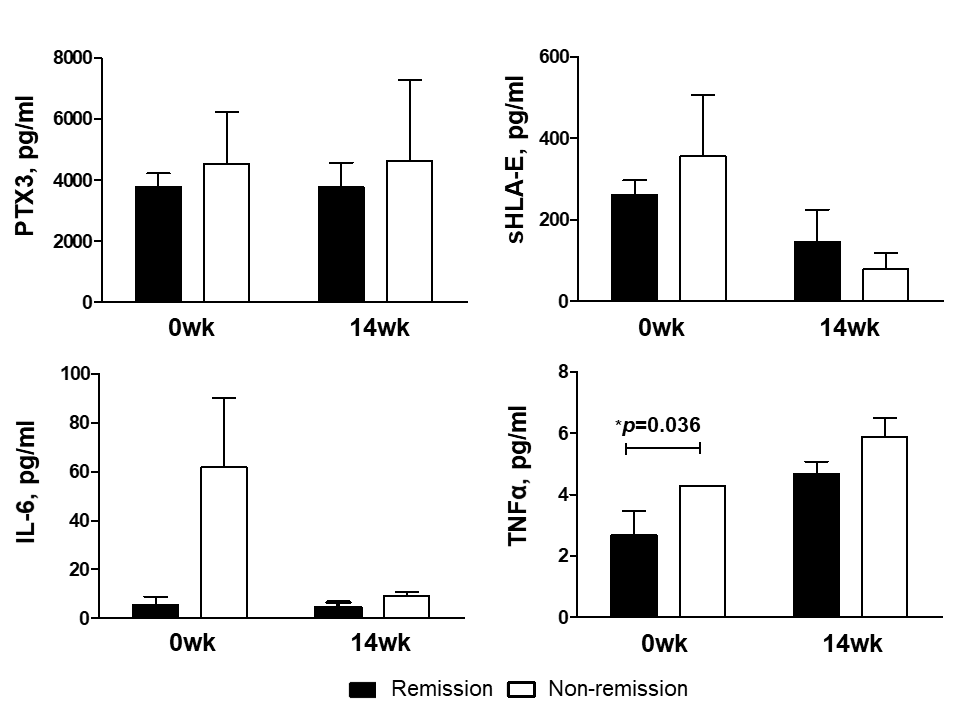
**

**Fig. 2 Serum levels of PTX3, sHLA-E, IL-6, and TNFα in patients with remission (n = 9) and non-remission (n = 2) at baseline and week 14.** Values are median and IQRs. PTX3, pentraxin-3; sHLA-E, soluble human leukocyte antigen-E; IL-6, interleukin-6; TNFα, tumor necrosis factor-α.
